# Recent Advances in Polymer of Intrinsic Microporosity (PIM) Membranes for Ion Separation Applications

**DOI:** 10.3390/membranes16040126

**Published:** 2026-03-31

**Authors:** Cuijing Liu, Jingyi Xu, Linbo Li

**Affiliations:** School of Metallurgical Engineering, Xi’an University of Architecture and Technology, Xi’an 710055, China; xujingyi@xauat.edu.cn

**Keywords:** polymers of intrinsic microporosity (PIMs), ion separation, ion resource recovery, water treatment, electrochemical energy storage

## Abstract

Polymers of intrinsic microporosity (PIMs) represent a novel class of microporous materials tailored for membrane separation. Initially explored predominantly for gas separation, they have subsequently found widespread utility in organic solvent nanofiltration. In recent years, their applicability has been further extended to ion separation. However, few comprehensive reviews have been dedicated to summarizing the advances of PIMs in this burgeoning field to date. This review provides a systematic overview of the recent progress in PIM membranes for ion separation. First, the structural features of PIMs employed in ion separation are summarized, with an emphasis on structure–performance correlations. Subsequently, their diverse applications in ion separation are elaborated in detail, encompassing ion resource recovery, water treatment, and electrochemical energy storage systems. Next, the current challenges facing the application of PIMs in ion separation are outlined, and finally, conclusions are provided. This review aims to provide insightful guidance for the development of high-performance PIM-based membranes in this rapidly evolving research area.

## 1. Introduction

Membrane technology offers distinct advantages over conventional separation processes, including facile continuous operation, a small footprint, and environmental benignity. Polymers of intrinsic microporosity (PIMs) are a class of amorphous microporous organic polymers, and PIM-1, the prototypical intrinsic microporous polymer introduced in 2004, has paved the way for the development of PIM-based microporous polymer materials [1]. These polymers are characterized by rigid, twisted and non-coplanar backbones that lack free rotation [2]. This structural feature prevents effective chain entanglement and packing, thereby facilitating the formation of continuous free volume within the microporosity range. Owing to their three-dimensional microporous architecture, PIMs have garnered considerable attention for the selective transport of gases, dyes, ionic species, etc.

Most studies on PIMs have centered on hydrophobic systems, with a primary focus on gas separation [3,4,5,6,7,8]. In recent years, PIMs have gained significant interest for liquid separation systems, mostly in organic solvent nanofiltration [9]. Comparatively, their applications in aqueous separations remain underexplored [10]. While several reviews mention the application of PIMs in ion separation [11,12,13,14], these sections are brief and lack comprehensive coverage of relevant ion separation fields. This review first elaborates on the structural characteristics of PIMs tailored for ion separation, and then comprehensively covers their diverse applications in ion separation. These applications are categorized into three main areas: (1) ion resource recovery, including acid/alkali recovery and lithium extraction from ion mixtures; (2) water treatment, further subdivided into reverse osmosis (RO) and nanofiltration (NF); (3) electrochemical energy storage, including redox flow battery (RFB), proton exchange membrane fuel cell (PEMFC), and anion exchange membrane fuel cell (AEMFC). Next, the current challenges associated with PIM membranes are discussed, and finally, conclusions are drawn.

## 2. PIMs Employed in Ion Separation

**Figure 1:**
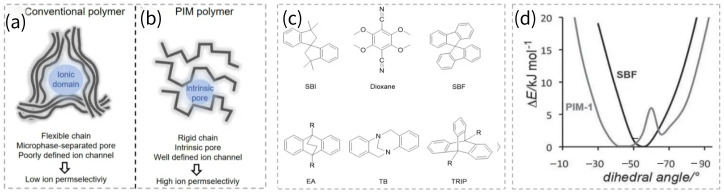
Schematic diagrams of membrane structure and ion permselectivity for (**a**) conventional polymers and (**b**) PIM polymers. (**c**) The commonly used building blocks in the PIM-based membranes are employed for ion separation. (**d**) Dihedral angles comparison of PIM-1 and SBF. Reproduced under CC BY license. Copyright 2016, Publisher Elsevier [19].

Ion permselectivity through a polymer membrane is regulated by transient free volume elements created by polymer dynamics [15]. Conventional membrane materials, such as polysulfone, poly (phenylene oxide) and polyphenylene, have high rotational freedom and can twist to maximize intra- or intermolecular interactions. The flexible chains pack without leaving obvious interchain pores, resulting in meager free volume [11]. Charged groups have been commonly introduced into the above polymers to form ion-conducting channels [16,17]. The resulting membranes possess complex microphase-separated structures, where ionic domains assemble into poorly defined ion channels and thus give rise to inferior ion separation performance [18]. In contrast, PIM membranes possess “frozen” free volume derived from their rigid, contorted polymer backbone. The pore structure can be precisely tailored through the rational design of rigid monomers, enabling well-defined pore channels that are beneficial for enhanced ion permselectivity. The differences in membrane structure and ion separation performance between conventional polymers and PIM polymers are illustrated in Figure 1a,b.

As illustrated in Figure 1c, PIMs can be engineered from sterically hindered monomeric building blocks, including spirobisindane (SBI), dioxane, spirobifluorene (SBF), ethanoanthracene (EA), Tröger’s base (TB), triptycene (TRIP), etc., which frustrate polymer chain packing to construct highly permeable and size-sieving microporous structures. The SBI and dioxane moieties are documented to possess relatively high flexibility. In contrast, SBF exhibits enhanced rigidity compared with SBI and dioxane segments in PIM-1, which is corroborated by the narrowed energy barriers of SBF (Figure 1d) [19]. Similarly, bridged bicyclic ring systems, such as EA and TB, which are increasingly adopted in the synthesis of next-generation PIMs, also demonstrate greater rigidity than SBI and dioxane rings [20]. Given the extensive utilization of PIM-1 and TB-based membranes in ion separations, these two representative materials are elaborated in detail in the subsequent sections.

### 2.1. PIM-1

**Figure 2:**
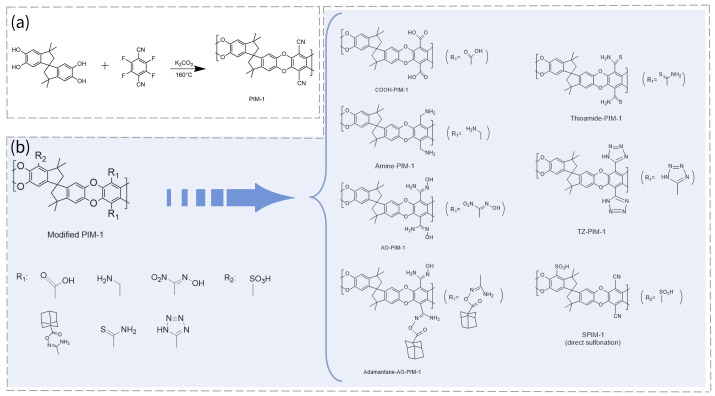
(**a**) Synthesis route of PIM-1. (**b**) The diverse strategies for the modification of PIM-1.

As the archetypal member of the PIM family, PIM-1 is also one of the limited number of PIMs that have been explored for deployment in hydrophilic systems via modification. As depicted in Figure 2a, PIM-1 can be synthesized by nucleophilic aromatic substitution between bis-catechol and fluorinated monomer [21]. However, the inherent hydrophobicity of pristine PIM-1 necessitates modifications to incorporate hydrophilic ionizable moieties, enabling more connected hydrophilic ion channels.

The diverse strategies for the modification of PIM-1 are illustrated in Figure 2b. First, carboxylic acid-functionalized PIM-1 (COOH-PIM-1) is obtained by hydrolyzing the cyano groups (-CN) of PIM-1 in an acidic environment, where -CN is converted to -COOH [22]. Next, amine-PIM-1 is prepared by stirring PIM-1 powder in anhydrous tetrahydrofuran (THF) under an inert atmosphere, cooling to 0 °C, and then adding 1.0 M borane-THF solution dropwise, followed by reflux overnight [23]. In addition, amidoxime-functionalized PIM-1 (AO-PIM-1) can be synthesized by dissolving PIM-1 polymer in THF, heating the solution to 65–70 °C under a nitrogen atmosphere, adding hydroxylamine dropwise, and then refluxing the mixture at around 69–70 °C for 20–24 h. After the reaction, the polymer is precipitated using ethanol, filtered, thoroughly washed with ethanol, and dried to obtain the final product [24]. Building on this, the adamantane-AO group is covalently grafted onto PIM-1 via a coupling reagent at room temperature in an organic solvent, ultimately forming adamantane-AO-PIM-1 [25]. The synthesis of thioamide-PIM-1 is achieved by reacting PIM-1 with phosphorus pentasulfide (P_2_S_5_) in the presence of sodium sulfite (Na_2_SO_3_), using a dioxane/ethanol solvent mixture under reflux conditions [26]. Furthermore, the synthesis of PIM-1 substituted with tetrazole groups (TZ-PIM-1) involves reacting PIM-1 as the starting material in Dimethylacetamide (DMAc) solvent at 120 °C with sodium azide (NaN_3_) and zinc chloride (ZnCl_2_) as catalysts for 8 days, leading to the formation of TZ-PIM-1 via an azide-nitrile cycloaddition reaction [27]. Finally, sulfonated PIM (SPIM) can be prepared by directly sulfonating PIM-1 using a sulfur trioxide/dichloromethane solution (SPIM-1) [28] or through copolymerization via Friedel–Crafts alkylation in a single step, where the sulfonated and non-sulfonated PIM monomers co-polymerize to produce SPIM [29]. All the aforementioned products can basically retain the intrinsic microporous structure of PIMs and can be tailored for diverse applications such as separation and electrochemical devices.

### 2.2. TB PIMs

**Figure 3:**
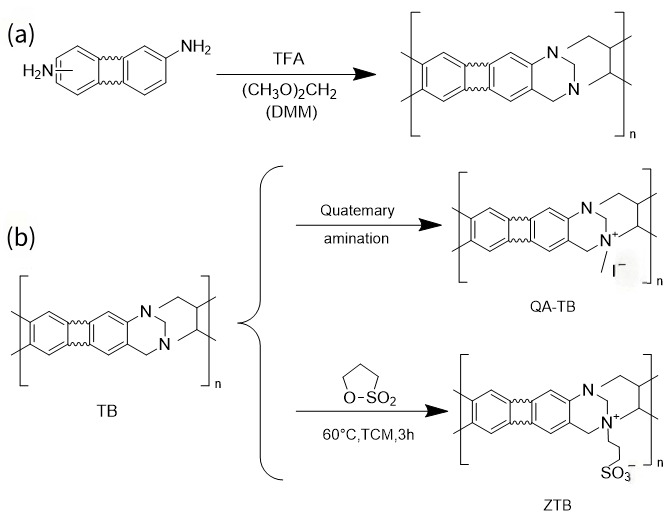
(**a**) Synthesis of TB polymers. (**b**) Quaternization and zwitterionization of TB group.

TB polymers represent a specific subclass of PIMs, characterized by the incorporation of 6H,12H-5,11-methanodibenzo[b,f][1,5]diazocine moieties. The predominant approach to synthesizing TB polymers involves the condensation reaction of diamine and formaldehyde precursors under strongly acidic conditions. After preliminary screening, researchers achieved the optimal synthetic performance by utilizing dimethoxymethane (DMM) as the formaldehyde source and trifluoroacetic acid (TFA) as the acidic medium (Figure 3a). Post-polymerization modification is also a viable strategy for tailoring the properties of TB-based PIMs (TB-PIMs), with the corresponding modification pathways illustrated in Figure 3b. The most extensively studied modification strategy involves the quaternization of bridged nitrogen atoms with electrophiles (e.g., methyl iodide), thereby introducing permanent positive charges on the nitrogen sites [30], creating a QA-TB membrane. Several such quaternized TB-PIMs are applicable for ion separation, with a detailed discussion to be presented subsequently. On the other hand, the zwitterionization of TB polymers (ZTB) can be achieved by reacting TB polymer with 1,3-propanesultone in trichloromethane (TCM) solvent [31].

## 3. Applications

**Figure 4:**
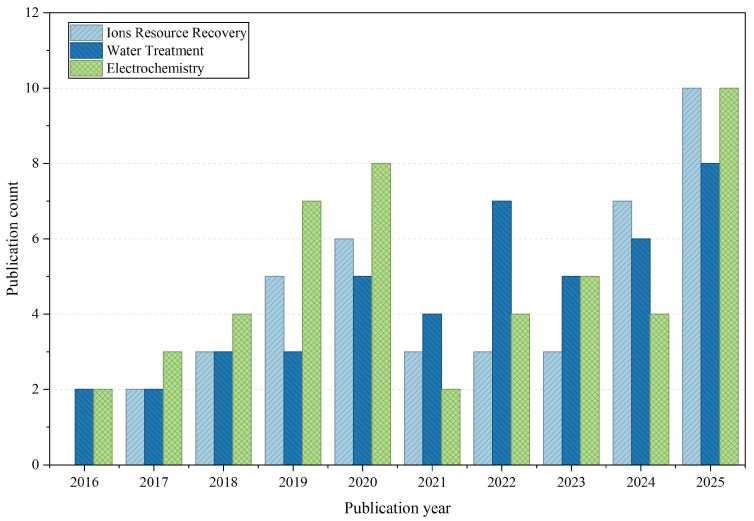
The annual number of published papers on “ion resource recovery”, “water treatment”, and “electrochemical energy storage” from 2016 to 2025. For “ion resource recovery”, the search was conducted using the research topics of “polymers of intrinsic microporosity membranes” and “acid recovery” or “alkali recovery” or “ion separation”. For “water treatment”, the search was conducted using the research topics of “polymers of intrinsic microporosity membranes” and “water treatment” or “nanofiltration” or “reverse osmosis”. For “electrochemical energy storage”, the search was conducted using the research topics of “polymers of intrinsic microporosity membranes” and “redox flow batteries” or “proton exchange membrane fuel cells” or “anion exchange membrane fuel cells”. All the searches were performed on Web of Science.

PIM membranes have garnered growing research attention for ion separation applications in recent years. Specifically, PIM membranes demonstrate remarkable utility across three core domains, including ion resource recovery, water treatment and electrochemical energy storage. For ion resource recovery, PIM membranes have been verified to enable the preferential transport of monovalent ions (e.g., H^+^, OH^−^, Li^+^) over multivalent ions, enabling efficient acid, alkali and Li^+^ recovery via diffusion dialysis or electrodialysis processes. In the realm of water treatment, PIM membranes are commonly employed in pressure-driven processes, including the RO process for seawater or brackish water desalination and the NF process for selective salt fractionation. Additionally, PIMs are making significant strides in electrochemical energy storage systems, particularly flow batteries and fuel cells. Their high ionic conductivity and superb ion/ion and ion/molecule selectivity facilitate the development of more efficient and durable energy storage systems, advancing renewable energy technologies. The annual publication output pertaining to these three major application fields is summarized in Figure 4. It demonstrates the growing attention devoted to PIM-based ion-separation membranes.

### 3.1. Ion Resource Recovery

#### 3.1.1. Acid/Alkali Recovery

Lin’s group introduced benzylic methyl groups into the PIM-1 polymer, followed by bromination of part of these groups via N-bromosuccinimide mediation or liquid bromine bromination, to synthesize bromomethylated polymers of intrinsic microporosity (PIM-Br) [21,32]. Subsequent crosslinking and cationization with 4,4′-bipyridine or 1,4-diazabicyclo[2.2.2]octane yielded membranes with acid recovery performance in a H^+^/Fe^2+^ system. The optimized membranes exhibit proton dialysis coefficients (U_H_^+^) of 51.07 × 10^−3^ m h^−1^ and 37.0 × 10^−3^ m h^−1^, with corresponding selectivities of 52.16 and 54.3, respectively.

**Figure 5:**
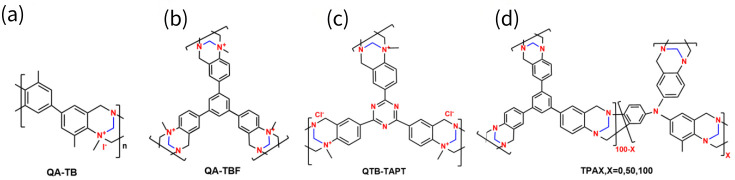
The molecular structures of the reported TB membranes used for ion separation: (**a**) QA-TB; (**b**) QA-TBF; (**c**) QTB-TAPT; (**d**) TPAX, X = 0, 50, 100.

TB membranes have also been reported to enable mono-/divalent ion separation, as summarized in Figure 5. The QA-TB membrane (Figure 5a) developed by Liu et al. was prepared by casting linear TB polymer and exhibited exceptional anion selectivity of 106 for Cl^−^/CO_3_^2−^ and 82 for Cl^−^/SO_4_^2−^, suggesting its efficient anion separation capability. Further advancements include an organic sol–gel strategy using trifunctional monomers to in situ synthesize crosslinked, swell-resistant, self-supporting TB membranes (QA-TBF, QTB-TAPT, TPAX membrane in Figure 5b–d) [33,34,35]. Lin’s group validated the high acid/alkali recovery performance of QA-TBF, with H^+^/Fe^2+^ and OH^−^/WO_4_^2−^ selectivities of 694.4 and 181.0, respectively [34]. Yang et al. developed a QTB-TAPT membrane containing triazine groups, which exhibited excellent acid recovery performance, with a U_H_^+^ of 22.80 × 10^−3^ m h^−1^ and a selectivity of 723.8, which are 2.7 times and 391.2 times higher than those of the commercial DF-120, respectively [35]. Liu et al. reported that tris(4-aminophenyl) amine, a smaller trifunctional monomer, can yield TB membranes (TPA100) with narrower pore sizes compared to those made from 1,3,5-tris(4-aminophenyl) benzene [36]. As a result, these TPA100 membranes demonstrated a high OH^−^/WO_4_^2−^ selectivity of 260.

#### 3.1.2. Lithium Resource Recovery

**Figure 6:**
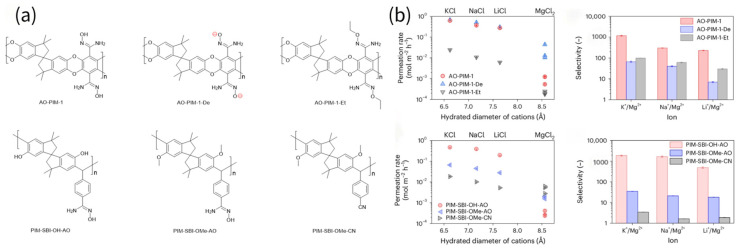
(**a**) The chemical structures of PIM membranes with different modified groups (adapted from Ref. [37]). (**b**) The mono-/divalent cations separation performance of comparison [37]. Reproduced under CC BY license. Copyright 2025, Publisher Nature Portfolio.

Yang et al. incorporated hydrophilic functional groups, including carboxylic acid, sulfonic acid and hydroxyl groups, into sub-nanometer-sized PIM polymers [37]. Carboxylic acid-, sulfonic acid-, or deprotonated hydroxyl group-modified PIM membranes were found to exhibit poor Li^+^/Mg^2+^ separation performance due to the negative membrane charge, which favors Mg^2+^ migration over monovalent ions. In contrast, hydroxyl group-modified PIM membranes demonstrated a superior ion permeation rate and ion selectivity. Compared to the ethoxy or non-hydroxyl group modifications, hydroxyl groups not only enhance local hydrophilicity to improve water channel connectivity but also facilitate interactions with ions and their transport. The related chemical structures are shown in Figure 6a, and AO-PIM-1 and PIM-SBI-OH-AO membranes outperformed their analogous counterparts (AO-PIM-1-De, AO-PIM-1-Et, PIM-SBI-OMe-AO, and PIM-SBI-OMe-CN). Figure 6b shows the detailed performance comparison of the above membranes. Impressively, PIM-SBI-OH-AO achieved a remarkable Li^+^/Mg^2+^ selectivity of up to 485, demonstrating exceptional ion discrimination capability.

Xu’s group synthesized a series of TB membranes for lithium resource recovery [38,39]. Dimethylbiphenyl (DMBP-TB), derived from the commercially available and cost-effective 4,4′-diamine-3,3′-dimethyl-biphenyl, has been extensively investigated. However, its inherent hydrophobicity renders the membrane impermeable to Li^+^. The acid protonation of the microporous DMBP-TB membrane affords a unique ion-conducting membrane, DMBP-TB^+^, demonstrating a Li^+^ permeation rate of 91.3 mmol m^−2^ h^−1^ and Li^+^/Mg^2+^ selectivity of 13.7 in a binary salt mixture. However, the protonated DMBP-TB^+^ may be deprotonated in water and basic solutions; therefore, it failed to reveal its full potential in ion separation. On the other hand, the quaternization of the DMBP-TB membrane resulted in a DMBP-QTB membrane, allowing marginal ion permeation, achieving a Li^+^ permeation rate of 2 mmol m^−2^ h^−1^, and a high Li^+^/Mg^2+^ selectivity of 152.2. However, the diffusion rates of monovalent cations (Li^+^, Na^+^) were reduced across the DMBP-TB^+^ membrane, which is probably due to its relatively low ion exchange capacity and decreased pore size after quaternization [40]. Furthermore, the dibenzo-18-crown-6 was incorporated into the TB skeleton as a Li^+^-selective moiety, yielding an improved Li^+^ permeation rate of 13 mmol m^−2^ h^−1^ and a moderate Li^+^/Mg^2+^ selectivity of 35.8 [38]. In addition, the same group incorporated dibenzo-15-crown-5 structural units into the rigid TB polymer skeleton and obtained a ^6^Li^+^/^7^Li^+^ selectivity of 1.08 [41]. As mentioned above, the TPA100 membrane, which was in situ produced from tris(4-aminophenyl) amine, demonstrated a 4.8-fold enhancement in Li^+^/Mg^2+^ selectivity (157 vs. 33) compared to the TPA0 membrane, due to its smaller pore size [36].

In addition, Gou et al. pioneered the creation of polyimide intrinsic microporous polymer (PIM-PI)-based artificial transmembrane lithium channels, suggesting their high Li^+^ selectivity over both K^+^ and Na^+^ [42,43]. Crown-ether-modified PIMs have been reported to be effective lithium extraction sorbents, which are also conductive to lithium resource recovery [44].

In general, PIM membranes exhibit favorable performance in acid/alkali recovery and lithium recovery, which is predominantly realized via diffusion dialysis or electrodialysis processes. To facilitate a clear comparison of the aforementioned membranes, key data on permeability and selectivity are summarized in Table 1.

### 3.2. Water Treatment

The micropore size of PIMs precisely matches the critical pore size range required for RO and NF membranes, which determines their substantial application potential as RO and NF membranes [15]. RO membranes are primarily engineered for seawater and brackish water desalination, where their nanoscale pore structures enable the efficient rejection of all ions. In contrast, NF membranes are tailored for selective divalent salt removal, characterized by their ability to retain multivalent ions (e.g., Ca^2+^, Mg^2+^, SO_4_^2−^) while permitting the partial passage of monovalent ions (e.g., Na^+^, Cl^−^), rendering NF membranes particularly suitable for applications such as water softening, wastewater reclamation, etc.

#### 3.2.1. RO

**Figure 7:**
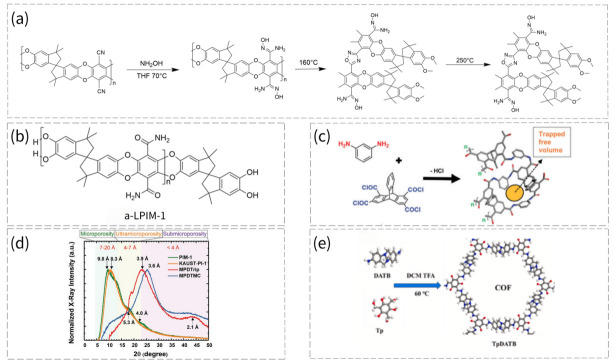
(**a**) Schematic of amidoximation reaction of PIM-1 and thermal treatment process of AOPIM-1 (adapted from Ref. [46]). (**b**) Molecular structure of water-soluble a-LPIM-1 (adapted from Ref. [48]). (**c**) Reaction scheme for the synthesis of crosslinked aromatic polyamide using MPD and triptycene-1,3,6,8-tetraacetyl chloride (Trip) monomers with internal free volume (IFV) trapped within chain repeat units [49]. Reproduced under CC BY license. Copyright 2020, Publisher Wiley. (**d**) XRD spectra of four PIM powders with average spacing obtained using Bragg’s law [49]. Reproduced under CC BY license. Copyright 2020, Publisher Wiley. (**e**) The synthesis of COF particles (TpDATB) [50]. Reproduced under CC BY license. Copyright 2026, Publisher Elsevier.

Despite the remarkable success of polyamide RO membranes in desalination, their lack of intrinsic pores hinders further water permeability improvement. PIMs offer a promising alternative. Shi et al. predicted via simulations that PIM-1 could outperform commercial RO membranes, but this study overlooked PIM-1′s hydrophobicity, which may largely hinder water permeation [45]. Yang et al. pioneered the use of PIMs by first modifying PIM-1 via amidoximation to fabricate a hydrophilic AOPIM-1 membrane, followed by post-thermal treatment to optimize its microstructure (Figure 7a) [46]. The fully amidoximized AO-PIM-1 membrane exhibited a 23-fold improvement in water permeability compared to unmodified PIM-1, suggesting a critical role of hydrophilic functionalization for PIM-based water treatment processes [47]. With a permeability of 1.92 × 10^−4^ LMH bar^−1^, AO-PIM-1 matches the performance of commercial BW30 and SW30 membranes. Notably, polyamide RO membranes typically possess crumpled surfaces, leading to intrinsic permeability far lower than measured values. Given AO-PIM-1′s comparable NaCl rejection (>98%) to polyamide membranes, its high permeability demonstrates significant potential for desalination.

PIM materials can also be used as new monomers or additives to polyamide layers for desalination performance improvement. Dou et al. synthesized a water-soluble a-LPIM-1 with low-molecular-weight and hydroxyl terminals as shown in Figure 7b and mixed it with m-phenylenediamine (MPD) as an aqueous monomer for interfacial polymerization [48]. Due to the twisted structure of a-LPIM-1 within the selective layer, the water permeance increased 2.1 times to 62.8 LMH bar^−1^ with acceptable NaCl rejection of 97.6%, when the added amount of a-LPIM-1 reached 0.5 wt%, without obviously compromising NaCl rejection. Ali et al. fabricated a polyamide layer, MPDTrip, using a bridge-bicyclic triptycene tetra-acyl chloride (Trip) as shown in Figure 7c [49]. Figure 7d compares the XRD spectra of MPDTrip with the other three microporous PIMs, and MPDTrip exhibited more tight chain packing than PIM-1. For water/salt separation, it showed high NaCl rejection around 96% with water permeance of 9.2 LMH bar^−1^. In addition, very recently, Wei et al. synthesized Covalent Organic Framework (COF) particles containing TB groups (TpDATB), using 3,3′-diamine-TB (DATB) and 1,3,5-triformylphloroglucinol (Tp) as monomers, as shown in Figure 7e [50]. Compared to the pristine polyamide membrane, the 8 wt% incorporation of COF particles into MPD aqueous solution led to a water permeance increase from 1.9 to 2.3 LMH bar^−1^, with a slight NaCl rejection from 99.64% to 99.58%. Notably, the modified RO membrane showed higher thermal resistance, achieving a high water permeance of 5.4 LMH bar^−1^ and a NaCl rejection of 99.1% even at 70 °C, probably due to the higher hydrogen bond crosslinking density.

#### 3.2.2. NF

Kim et al. pioneered a strategy to fabricate a carbonaceous NF membrane (PC-PIM-1) with sub-nanometer interconnected pores, via a combination of controlled carbonization of PIM-1 membranes and O_2_ plasma treatment [51]. The resulting pores facilitated water permeation, yielding high water permeability of 13.30 LMH bar^−1^, a high MgSO_4_ rejection of 77.38%, and favorable antifouling performance. Jeon et al. prepared four sub-nanometer membrane materials also using carboxyl-functionalized intrinsic microporous polymer (PIM-COOH) as a precursor through carbonization at different temperatures [52], including the original polymer membrane (PIM-COOH), thermally decarboxylated crosslinked membrane at 350 °C (cPIM-COOH 350), amorphous carbon membrane at 600 °C (cPIM-COOH 600), and graphitized carbon membrane at 1100 °C (cPIM-COOH 1100). Experimental results demonstrated that with increasing carbonization temperature, the water flux of the membranes increased from 0.86 LMH bar^−1^ for PIM-COOH to 3.83 LMH bar^−1^ for cPIM-COOH 1100, while maintaining high MgSO_4_ rejections above 80%.

In addition, incorporating monomers with rigidly contorted structures into the polyamide layer can construct additional water transport channels, thereby mitigating the permeability–selectivity trade-off inherent to conventional membranes. Tang et al. employed interfacial polymerization to synthesize a selective polyamide layer by using 5,5,6′,6′-tetrahydroxy-3,3,3′,3′-tetramethyl spirobisindane (TTSBI) and piperazine (PIP) as aqueous-phase comonomers [53]. TTSBI regulated monomer diffusion and reaction rates, inducing a distinctive crater-like surface morphology. This increased the effective permeable area and elevated water permeability to 9.9 LMH bar^−1^, which is twice that of conventional membranes, while maintaining a Na_2_SO_4_ rejection rate of 98%. Wang et al. designed β-cyclodextrin-modified hydrophilic PIM nanoparticles (β-CD-PIM NPs) and utilized them as aqueous-phase additives for polyamide layer preparation [54]. The incorporation of β-CD significantly enhanced the hydrophilicity of PIMs, and its microporous structure served as an efficient water transport channel. The resulting membrane achieved a water permeability that was three times higher than that of the original membrane and a Na_2_SO_4_ rejection rate of 95.1%.

In terms of dye separation, Ye et al. successfully developed a composite membrane material by using carbon nanotube films as a substrate, growing a dense UiO-66 layer, and coating the surface with an intrinsically microporous polymer (PIM-1-coated UiO-66) [55]. This method achieved efficient separation of multiple dyes, with a separation efficiency exceeding 90%. The separation mechanism primarily relies on the synergy of sieving effects and electrostatic interactions, resulting in outstanding dye separation performance. Dou et al. prepared polyarylate nanofiltration membranes similar to PIMs by using TTSBI with a distorted structure and three acyl chlorides, including trimesoyl chloride (TMC), short-chain glutaryl chloride (GC) and long-chain sebacoyl dichloride (SDC) through interfacial polymerization, achieving high flux and tunable dye/salt separation performance [56]. Among them, the TTSBI-TMC membrane exhibited a water flux of 480.5 LMH bar^−1^ (nearly seven times higher than that of conventional membranes), while the TTSBI-GC membrane achieved balanced performance with a dye rejection rate of 95.4%, a salt rejection rate of 8.3%, and a water flux of 402.4 LMH bar^−1^.

**Figure 8:**
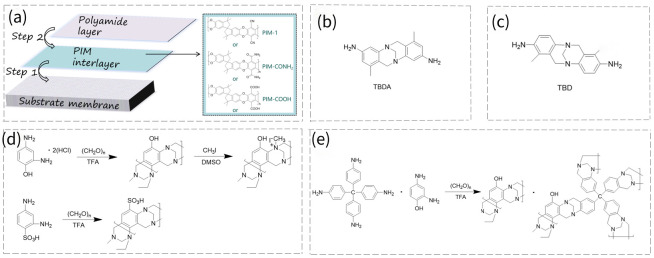
(**a**) The fabrication of NF membranes with PIM interlayers (adapted from Ref. [57]). (**b**) Chemical structure of TBDA monomer (adapted from Ref. [59]). (**c**) Chemical structure of TBD monomer (adapted from Ref. [62]). (**d**) Chemical structures of quaternized and sulfonic acid functionalized TB PIMs (adapted from Ref. [10]). (**e**) Chemical structure of crosslinked TB polymer (adapted from Ref. [65]).

Using PIMs as interlayers during interfacial polymerization has also been proven to be an effective strategy to modulate membrane performance. Tang et al. employed PIM-1 and its derivatives (PIM-CONH_2_, PIM-COOH) as interlayers for NF membrane fabrication, as illustrated in Figure 8a [57]. A pristine membrane without a PIM interlayer, PA, showed a water flux of 13.8 LMH bar^−1^ and a Na_2_SO_4_ rejection of 99.3%. In contrast, a M-3 membrane with a PIM-1 interlayer exhibited a remarkable increase in water flux (24.2 LMH bar^−1^), while retaining a high Na_2_SO_4_ rejection of 98.5%. A M-N membrane with an incorporated PIM-CONH_2_ interlayer achieved an even higher water flux of 28.2 LMH bar^−1^ with 94.5% Na_2_SO_4_ rejection. The M-C membrane with a PIM-COOH interlayer delivered a water flux of 21 LMH bar^−1^ and 95.0% Na_2_SO_4_ rejection. These variations were attributed to the different functional groups, which modulated hydrophilicity, surface charge, and morphology of the selective layer.

Due to the unique V-shaped, rigid, twisted geometry of TB, it can effectively disrupt the tight packing of polymer chains and induce the formation of a microporous structure [58]. Therefore, monomers containing TB groups have also been integrated into polyamide layers. Liu’s group developed several NF membranes using the TB-containing diamine monomer (2,8-diamino-4,10-dimethyl-6H,12H-5,11 methanodibenzo [1,5]-diazocine (TBDA) for polyamide layer construction, and the structure of TBDA is illustrated in Figure 8b [59]. Given the poor water solubility but good dimethyl sulfoxide (DMSO) solubility of TBDA, the monomer was incorporated into the DMSO casting solution of polyethersulfone (PES). Interfacial polymerization was then performed directly on this PES substrate. The developed polyamide membrane exhibited a water permeance of 5.4 LMH bar^−1^ and a Na_2_SO_4_ rejection of 93.8%. In another approach, TBDA and PIP were co-dissolved into the DMSO casting solution of PES, followed by reaction with TMC. The membrane delivered a water permeance of 14.4 LMH bar^−1^ and a Na_2_SO_4_ rejection of 99.3% [60]. Gong et al. added DATB into a PES solution to form PES membranes, followed by interfacial polymerization to form a DATB-based NF membrane [61]. The optimized NF membrane exhibited a lower water permeance (6.3 LMH bar^−1^ vs. 9.7 LMH bar^−1^) and a higher Na_2_SO_4_ rejection (98.1% vs. 93.8%) than the conventional PIP-TMC NF membrane. This probably resulted from the reduced crosslinking density of the PIP-TMC NF membrane due to the higher PIP loss during the PES membrane formation process. The DATB-based NF membrane had especially excellent acid resistance. Lee et al. fabricated a structurally analogous TB-containing diamine monomer, Tröger’s base diamine (TBD), as depicted in Figure 8c [62]. Benefiting from the good solubility of TBD in acidic water, a conventional interfacial polymerization process was employed to construct a TBD-TMC polyamide layer. Relative to the benchmark MPD-TMC membrane, the TBD-TMC membrane achieved a 570% enhancement in water permeance, along with a Na_2_SO_4_ rejection of 91% and high monovalent/divalent ion selectivity (~7.0 for NaCl/Na_2_SO_4_ separation). In addition, the TBD-TMC membrane showed a remarkably high water permeance of 17.0 LMH bar^−1^. Liu et al. [63]. dissolved a TB monomer in a water/ethanol solution containing PIP to prepare an aqueous-phase solution, which was subsequently reacted with a TMC/hexane solution. The addition of TB in the aqueous phase accelerated PIP diffusion and acted as a catalyst to boost reaction activity, thus forming a thinner polyamide layer (TB-PA). The water permeance of 18.5 ± 1.4 LMH bar^−1^ is twice that of the membrane without TB additive, while retaining a similar Na_2_SO_4_ rejection rate of 98.3%. Notably, the same monomer was verified to enhance the microporosity of the polyamide layer, affording high methanol permeability as well. In addition. Lu et al. fabricated membranes from TTSBI and isophthaloyl chloride (IPC) [64]. The membrane exhibited high water permeability of 250 LMH bar^−1^, but a low NaCl rejection rate of ~10%, highlighting the high potential for dye/salt separation.

TB polymers can also be directly used to fabricate NF membranes. Agarwal et al. synthesized quaternized and sulfonic acid-functionalized TB PIM polymers, as shown in Figure 8d [10]. The two oppositely charged polymers were assembled into an NF membrane via a layer-by-layer method. The resulting membrane exhibited a water flux of 5–6 LMH bar^−1^ with the PEG rejection of 95% above the molecular weight of 500 Da. The same group further synthesized a crosslinked TB polymer as shown in Figure 8e [65]. After triglycidyl ether (TGE) crosslinking, the resulting NF membrane showed a water flux of 0.3 LMH bar^−1^ and a CuSO_4_ rejection of 87%. This work expands the library of polymers that could be utilized as selective active layers and provides a simple and scalable route to fabricate NF membranes from TB PIMs. It is noted that TB polymers can also be fabricated as ultrafiltration membranes by the non-solvent induced phase inversion method [66].

PIM-based RO and NF membranes exhibit high water permeability and salt rejection. Based on the above discussion, the performances of PIM membranes in RO and NF processes are summarized in Table 2.

### 3.3. Electrochemical Energy Storage

#### 3.3.1. RFB

Reported active species in RFBs include metal-based, organic-based, and inorganic non-metallic-based active compounds. Metal-based active species mainly encompass vanadium-based electrolytes employed in vanadium redox flow batteries (VRFBs), iron-based substances utilized in iron/chromium or iron/vanadium RFBs, zinc-based substances applied in zinc/bromine or zinc/iron RFBs, and ferricyanide complexes (e.g., K_4_Fe(CN)_6_) for ferricyanide-based RFBs. Organic-based active substances consist of quinones (e.g., 2,5-dihydroxy-1,4-benzoquinone (DHBQ), 2,6-dihydroxyanthraquinone (2,6-DHAQ), 2,6-di-2-propionate ether anthraquinone (2,6-D2PEAQ)) and flavin-based bioactive compounds (e.g., riboflavin 5’-phosphate sodium salt (FMN-Na), flavin adenine dinucleotide disodium salt hydrate (FAD-Na)). Inorganic non-metallic-based active compounds cover halogens, sulfur, etc. Membranes employed in RFBs must minimize the crossover of large redox-active species while enabling the fast transport of small charge-carrier ions [67]. Commercial perfluorosulfonic acid (PFSA) membranes, such as Nafion, are expensive and suffer from swelling issues. In addition, the production of PFSA involves the use of polyfluoroalkyl substances, known as “forever chemicals” [68]. Efforts have been devoted to the development of novel membranes, and PIMs have been found to be promising alternatives.

Chae et al. reported the high proton/vanadium selectivity of PIM-1; however, due to its hydrophobicity, it exhibited high membrane resistance [69]. Hydrophilic modifications can facilitate the formation of water channels. Baran et al. modified the PIM membrane with AO functionalities, exhibiting 0.06% capacity loss per cycle with Coulombic efficiencies steady at 99%, which is much lower than with Nafion 212 and Celgard 3501, in a Zn/TEMPO-sulfate battery [70].

Song’s group did a series of related studies. They compared PIM-1 with its hydrophilic derivative AO-PIM-1 [67]. Despite the material having the higher specific surface area (778 m^2^ g^−1^) and larger micropore volume (0.251 cm^3^ g^−1^), PIM-1 exhibited a relatively low water vapor adsorption capacity (5.7 wt%). In contrast, for the lower-porosity AO-PIM-1, the water vapor uptake increased linearly from 5.7 to 29.0 wt% as the AO functionalization content was adjusted from 0 to 100%. The ideal selectivity of K^+^ over Mg^2+^ and the larger Fe(CN)_6_^3−^ was up to around 35 and 82, respectively, for the AO-PIM-1 membrane. An AO-PIM-1 membrane was incorporated into aqueous RFBs using two kinds of couples, FMN-Na|K_4_Fe(CN)_6_ and 2,6-DHAQ|K_4_Fe(CN)_6_. Crossover tests demonstrate near 100% rejection of FMN-Na and 2,6-DHAQ by the membrane. The crossover rate of iron-containing species through AO-PIM-1 was significantly lower than that of an identical RFB using a Nafion 212 membrane. In a FMN-Na|K_4_Fe(CN)_6_ RFB, the effective blockage of active species confers high cycling stability with an electrochemical capacity retention of 84.5% over 200 cycles at 80 mA cm^−2^. This performance is comparable to that achieved with a Nafion 212 membrane in an equivalent RFB (85.1%). An RFB based on the 2,6-DHAQ|K_4_Fe(CN)_6_ redox couple in an argon-filled glovebox achieved a high coulombic efficiency of >99.8% with an AO-PIM-1 membrane at 40 mA cm^−2^. The RFB using an AO-PIM-1 membrane exhibited the lowest capacity fade rate of 0.5% per day, which is superior to the performance of a Nafion 212 membrane (2.3% per day).

**Figure 9:**
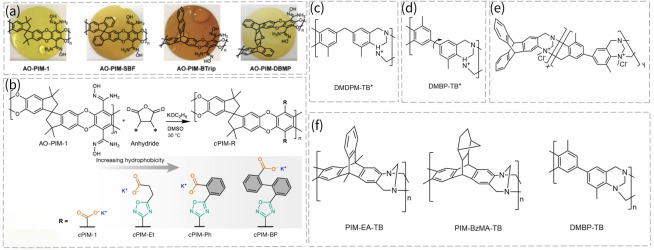
(**a**) Chemical structures of SBI, SBF, BTrip and DBMP units in AO-PIMs membranes [68]. Reproduced under CC BY license. Copyright 2022, Publisher Wiley. (**b**) Structural modification of AO-PIM-1 membrane via adjusting aromatic rings on charged groups and synthesis of cPIM derivatives [18]. Reproduced under CC BY license. Copyright 2024, Publisher Nature Portfolio; chemical structure of (**c**) DMDPM-TB^+^. (**d**) DMBP-TB^+^ (adapted from Ref. [39]). (**e**) The cross-linked structure of TB membrane copolymerized by DMBP and 2,6(7)-diaminotriptycene (Trip-NH_2_) (adapted from Ref. [72]). (**f**) Structures of PIM-EA-TB, PIM-BzMA TB and DMBP-TB (adapted from Ref. [67]).

Furthermore, Song’s group chose four units, SBI, SBF, benzotriptycene (BTrip) and dibenzomethanopentacene (DBMP), as shown in Figure 9a, to construct AO-PIM membranes [68]. Compared to Nafion 212, the four membranes displayed higher selectivities for KOH/redox species and mono-/divalent cations. The RFB assembled with the redox couple 2,6-DPPAQ|K_4_Fe(CN)_6_ employing an AO-PIM-DBMP membrane delivered a capacity retention of 98.78% over 1000 cycles at 80 mA cm^−2^, which is superior to those of Nafion 212 and Nafion 115 membranes, with retentions of 76.20% and 93.87%, respectively. This long-term stability was due to reduced crossover, the alleviation of redox molecule adsorption when using AO-PIM membranes, and improved redox molecule stability at a near-neutral pH value.

Charge-neutral PIMs retain pore dimensions but exhibit low ionic conductivity. Introducing charged moieties usually causes pore swelling, impairing selectivity. Creating narrow gates connecting micropores can break the conductivity–selectivity trade-off. Song’s group further proposed tailoring the local hydrophobic environment around ion-conducting channels in an AO-PIM-1 membrane by adjusting the number of aromatic rings attached to charged groups [18]. Four membranes (cPIM-1, cPIM-Et, cPIM-Ph, and cPIM-Bp) were compared (Figure 9b), with cPIM-Ph exhibiting the best conductivity–selectivity balance. When incorporated into RFBs using 2,6-D2PEAQ|ferrocyanide as the redox couple, cPIM-Ph achieved an ultralow capacity decay rate of 0.014% per day, and its coulombic efficiency reached 99.9%, which was 100 and 10 times lower than that of the benchmark Nafion 212 and sPEEK membranes, respectively.

PIM polymers can also be mixed with conventional polymers for performance enhancement. Hou et al. constructed a rigid P/cPIM membrane by incorporating rigid carbonylated PIM-1 (cPIM) chains into flexible PFSA chains [71]. The polarity of PIM-1 grafted with -COOH groups was significantly elevated, thus vastly increasing their mutual solubility in a PFSA solution. The mechanical interlocking of rigid chains restricts PFSA chain mobility, reducing membrane swelling and enhancing ion selectivity. The P/cPIM membrane exhibited an I_3_^−^ permeability one order of magnitude lower than that of the PFSA membrane. In a polysulfide/polyiodide redox flow battery (PSIB) test, when the state of charge (SOC) was elevated to 80%, the P/cPIM membrane maintained stable operation for over 780 h without capacity degradation, and the coulombic efficiency could reach 98%, significantly higher than the 165 h of the PFSA membrane. Meanwhile, the electrochemical capacity retention rate was maintained at 99.99%.

TB-based membranes also act well in this field. Xu’s group developed highly conductive and vanadium-sieving microporous TB membranes for VRFBs [39]. The rigid DMBP-TB exhibited a higher BET surface area than that of DMDPM-TB made from a dimethyldiphenylmethane (DMDPM) monomer. The peak power densities at ~100% SOC of protonated DMDPM-TB^+^ and DMBP-TB^+^ cells are 520.0 and 710.9 mW cm^−2^, respectively, which are higher than those of Nafion 117 cells (482.5 mW cm^−2^). Furthermore, DMDPM-TB^+^ and DMBP-TB^+^ maintained electrochemical capacity retention rates of 99.90% and 99.81%, respectively, after 100 cycles, with coulombic efficiency approaching 100%. The chemical structures of the two membranes are shown in Figure 9c,d. The same group copolymerized DMBP with 2,6 (7)-diaminotriptycene (Trip-NH_2_), and the latter had a higher rigidity; the resulting membrane structure is shown in Figure 9e [72]. By changing the monomer ratio, a series of TB membranes with varying BET surface areas but similar pore size distributions was obtained. The membrane with a Trip-NH_2_ ratio of 100% exhibited the highest power, and the membrane with a Trip-NH_2_ ratio of 80% stands out among all membranes in a pH-neutral RFB with FcNCl|BTMAP-Vi as the redox couple [59]. To step further, the same group in situ synthesized and quaternized a crosslinked TB membrane called Q-TBF-TPB [33]. The BTMAP-Vi permeability of this membrane was lower than that of many linear PIMs and commercially available membranes (Selemion DSV, Fumasep FAA-3-PE-30). The combination of fast ion conductivity and high selectivity enabled it to have a stable Coulombic efficiency of over 99% and a stable energy efficiency of 72.5% in BTMAP-Vi|TEMPTMA-based neutral RFBs over 1000 cycles. Song’s group also developed three kinds of TB membranes (PIM-EA-TB, PIM-BzMA-TB and DMBP-TB), as exhibited in Figure 9f [67]. It was found that PIM-EA-TB had the highest surface area, while DMBP-TB had the lowest surface area. PIM-EA-TB provided the highest K^+^ permeation rate of around 10 mol m^−2^ h^−1^, and a K^+^/Mg^2+^ selectivity of 15. PIM-EA-TB performed well in RFBs based on the 2,6-DHAQ|K_4_Fe(CN)_6_ and on FMN-Na|K_4_Fe(CN)_6_ couples. In terms of battery performance, PIM-EA-TB demonstrated high cycling stability with an electrochemical capacity retention of 86.5% over 200 cycles at 80 mA cm^−2^, and its Coulombic efficiency can reach 99.8% at 80 mA cm^−2^.

#### 3.3.2. PEMFC

Compared to the low-temperature PEMFC, operated around 80 °C, high-temperature proton exchange membrane fuel cells (HT-PEMFCs) operated at 100–200 °C hold great commercial potential for electrochemical devices owing to their easy water/heat management and enhanced reaction efficiency [73]. HT-PEMs play a pivotal role in isolating electrode reactions, supporting ion-carrier loading, and facilitating fast ion transport. Phosphoric acid (PA)-doped polybenzimidazole (PBI)-based membranes (e.g., PA-doped m-PBI membranes) are regarded as the state of the art in HT-PEMs due to their favorable proton conductivity under high-temperature and low-humidity conditions. However, the compact structures of PBI-based polymers result in small fractional free volumes, which are not conducive to the adsorption and retention of proton carriers.

**Figure 10:**
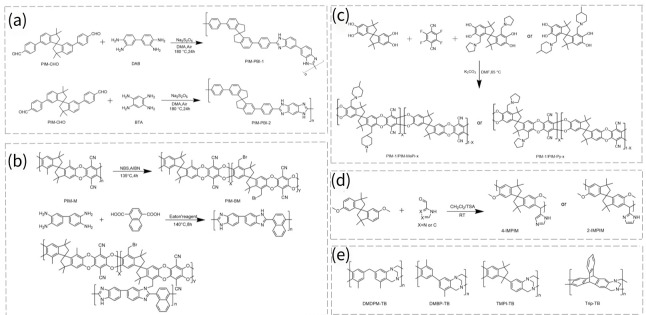
(**a**) Synthesis of PIM-PBIs and preparation of composite membranes (adapted from Ref. [75]). (**b**) Synthesis route of PIM-BM crosslinked PBI membranes (adapted from Ref. [76]). (**c**) Functionalization of PIM-1 via Mannich reaction (adapted from Ref. [77]). (**d**) Structural design of imidazole-doped PIMs membranes (adapted from Ref. [78]). (**e**) Structures of four different TB polymers (adapted from Ref. [79]).

PIMs exhibit good potential in HT-PEMFCs due to their tunable microporosity and functionality. PIMs can act as additives to enhance conventional polymers’ (e.g., m-PBI membranes) performance. Wang et al. incorporated PIMs into an aryether-type PBI (OPBI) matrix to form alloy membranes and observed the effect of the PIMs’ molecular weights on the properties of the PBI/PIM alloy membranes [74]. The alloy membranes achieved good H_2_/O_2_ cell performance. The peak power density of an L-10 PA-doped membrane reached 438 mW cm^−2^ at 160 °C without humidification, the proton conductivity of OPBI/L-PIM-10 was 313 mS cm^−1^, and the retention rate of PA was 65.4%. Jin’s group synthesized two kinds of PBI-based PIMs (PIM-PBIs), as shown in Figure 10a, and embedded PIM-PBIs in polyvinylidene fluoride PVDF to form mixed-matrix membranes for high-temperature proton conduction [75]. The resulting membranes exhibited high proton conductivity of up to 90.11 mS cm^−1^ at 140 °C and a high PA retention rate of 90.0%. Guo et al. proposed using bromomethylated PIM (PIM-BM) as a macromolecular crosslinker to tune the microporous structure of PBI HT-PEM [76]. The chemical synthesis routes for PIM-BM, PBI polymers, and their crosslinked products are shown in Figure 10b. The PIM-BM crosslinked PBI membrane displayed higher PA retention than the PBI membrane. The cell of an optimized membrane (NPBI/PIM-BM-15/PA) achieved a peak power density of 565 mW cm^−2^ at 160 °C, with a proton conductivity of 313 mS cm^−1^, and a retention rate of PA of 92.0%.

PIM polymers can also be directly used for HT-PEMs without blending with conventional polymers. Li’s group introduced alkaline pyrrolidine and piperidine functional groups onto PIM-1 via the classical Mannich reaction, obtaining PIM-1/PIM-Py-50 and PIM-1/PIM-MePi-50, as shown in Figure 10c [77]. Compared with commercial Meta-polybenzimidazole (m-PBI) membrane, PIM membranes exhibited superior PA retention and conductivity due to their grafting of alkaline groups and rich micropores. The proton conductivity and proton retention rates of the two materials were 74.13% and 68.52%, respectively, with peak proton conductivities of 42.02 mS cm^−1^ and 37.11 mS cm^−1^. Their peak power densities were 355 mW cm^−2^ and 295 mW cm^−2^, respectively. Zhou et al. first reported on the imidazole groups incorporated into PIM membranes, as shown in Figure 10d [78]. The combination of alkaline nitrogen heterocycles and inefficient chain packing enabled the formation of membranes with better PA retention ability than that of m-PBI membranes due to their strong acid–base interactions and capillary force. The proton conductivities at 180 °C of the 2-CIMPIM membrane and the 4-CIMPIM membrane were 330.3 mS cm^−1^ and 157.4 mS cm^−1^, respectively, which were obviously higher than those of PA-doped m-PBI (60 mS cm^−1^ for an acid PA doping level of 7). The superiority of the proton conductivity of 2-CIMPIM over 4-CIMPIM was attributed to the higher acid doping level of 2-CIMPIM.

Tang et al. fabricated four TB-based membranes with varied intrinsic ultramicropores for PA-doped proton exchange membranes, including DMDPM-TB, DMBP-TB, trimethylphenylindan (TMPI-TB) and Trip-TB, as shown in Figure 10e [79]. DMDPM-TB, with the best chain flexibility, was soluble in 85% PA, which was not suitable for PEM applications. The micropores of DMBP-TB and TMPI-TB membranes can be filled with PA, while for the Trip-TB membrane, the micropores with a small pore radius of 2.8 Å were unoccupied by PA. DMBP-TB exhibited the highest PA doping amount (72.5%) and acid-doping-level retention after water washing. The PA-doped DMBP-TB displayed 95% peak power density retention after 150 start-up/shut-down cycles at 15 °C and could accomplish over 100 cycles, even at −20 °C. In addition, the rigid bicyclic tertiary amines in TB-based membranes appeared to be critical motifs for PA absorption, because a low PA uptake of 5% was observed for the PIM-1 membrane. Wang et al. found that the DMBP-TB membrane made from polymers with a lower molecular weight had a higher PA content [80]. They used DMBP-TB polymers with 50 KDa, which was lower than the 99 kDa used in Tang’s work. The acid doping content of DMBP-TB reached a highest value of 1077%; however, the DMBP-TB/1077%PA membrane suffered from poor mechanical stability owing to the PA plasticization effect. Tang et al. combined DMBP-TB and poly (ether ketone cardo) (PEKC) to make a blend membrane. The resulting DMBP-TB/50%PEKC/149%PA membrane displayed an anhydrous conductivity of 0.061 S cm^−1^ at 180 °C, which enabled the creation of an H_2_–O_2_ fuel cell with a peak power density of 536 mW cm^−2^ at 180 °C without backpressure, suggesting its utility for HT-PEMFC applications, and its proton conductivity was 61 mS cm^−1^.

#### 3.3.3. AEMFC

PEMFCs are the most rapidly developing energy-conversion device in the 21st century. However, the acidic environment of PEMFCs limits the choice of catalysts to expensive noble metal catalysts, which hinders their large-scale development. AEMFCs offer an alkaline environment, in which the kinetics of oxygen reduction are enhanced, so the catalysts are no longer limited to just noble metals, reducing costs [81]. Anion exchange membranes (AEMs) serve as a pivotal component for AEMFCs, acting both as the electrolyte matrix to mediate ion migration between the cathode and anode and a barrier to inhibit the crossover of fuel and oxidizing agent. For AEMFCs to deliver sustained high-power output, AEMs must possess high hydroxide conductivity combined with excellent stability. A favorable trade-off between conductivity and stability is more readily attainable with a relatively low ion exchange capacity (IEC), as this design also helps to reduce the ion transport resistance across the membrane [82].

Ishiwari et al. fabricated PIM-based AEMs carrying trimethylammonium hydroxide groups, which exhibited a moderate OH^−^ conductivity of 65 mS cm^−1^ at 80 °C under 100% relative humidity [83]. High rigidity usually causes the brittleness of PIM AEMs, which can be alleviated by increasing the molecular weight of the PIM polymers. Guiver’s group reported a mechanically robust and highly anion-conductive PIM AEM (QPIM-1) membrane, fabricated by facile animation and quaternization of a PIM-1 polymer with a high molecular weight of ~184 kDa [84]. A high OH^−^ conductivity of 57 mS cm^−1^ at 20 °C was obtained, which was 2.6–5.3 times higher than that of dense QPPO AEM at similar IECs. Furthermore, the same group investigated hydrogen crossover through QPIM-1 AEMs under different hydration states, with the aim of enhancing fuel cell performance [85]. A prominent water-blocking effect on hydrogen crossover was detected, as indicated by a marked reduction in H_2_ permeability when dry AEMs were subjected to humidified gas streams. Hydrogen crossover in QPIM-1 AEMFC was suppressed to a low level at 60% relative humidity (RH) and remained low at higher RH levels. This phenomenon illustrates the favorable synergistic effect arising from rigid micropores that mitigate plasticization, combined with water occupation of micropores. In situ open circuit voltage of a QPIM-1 AEM-based fuel cell reached high values of 0.975 V at 100% RH and 0.925 V at 40% RH, indicating minimal hydrogen crossover across a broad RH range. Gong et al. fabricated a novel block copolymer AEM, which contained hydrophobic PIM blocks and hydrophilic, quaternized polysulfone blocks [82]. The PIM block imparts high free volume, and the hydrophilic block can self-assemble to a hydrophilic microphase, both of which are favorable for OH^−^ transport. The membrane exhibited a conductivity of 52.6 mS cm^−1^ at 80 °C with a relatively low IEC of 0.91 mmol g^−1^. The assembled H_2_/O_2_ fuel cell yielded a peak power density of 270 mW cm^−2^ at 560 mA cm^−2^.

**Figure 11:**
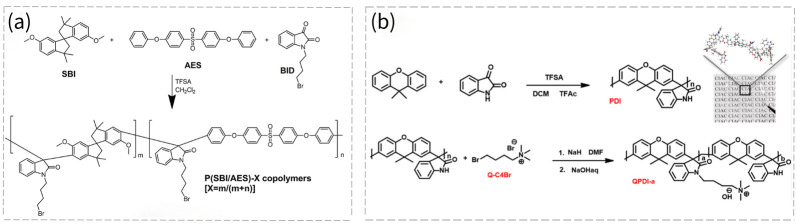
(**a**) Synthesis route of P(SBI/AES)-X copolymers [81]. Reproduced under CC BY license. Copyright 2020, Publisher ACS. (**b**) Synthesis route of QPDI-a AEMs [86]. Reproduced under CC BY license. Copyright 2020, Publisher Nature Portfolio.

Even though the above studies have been reported, the synthesis of PIMs requires the preparation of purified polymers, and some of the synthesis conditions are difficult to supply. Moreover, it is worth noting that some PIM-based AEMs contain unstable structures under alkaline conditions [86]. The intrinsic pore structure applied in the AEMs is scarce, and novel membranes and synthesis methods are still needed. Li et al. proposed a novel synthesis method to incorporate SBI groups into AEMs by the superacid-catalyzed polyhydroxyalkylation reaction [81]. They used flexible 4,4′-sulfonylbis(phenoxybenzene) (AES), rigid 6,6′-dimethoxy-3,3,3′,3′-tetramethyl-1,1′-spirobisindane and 1-(4-bromobutyl)-indoline-2,3-dione (BID) as monomers, and formed P(SBI/AES)-X copolymers, in which X represented the SBI monomer content. The reaction is shown in Figure 11a. After quaternization, QP(SBI/AES)-X AEMs can be obtained. The QP(SBI/AES)-0.5 AEM exhibited a high OH^−^ conductivity of 110 mS cm^−1^ at 80 °C with an IEC of 1.59 mmol g^−1^, as well as good mechanical robustness. Meanwhile, it yielded a maximum power density of 437 mW cm^−2^ at a current density of 1052 mA cm^−2^, 115% higher than QP(SBI/AES)-0. Zhang’s group used the same superacid-catalyzed reaction, as shown in Figure 11b, to synthesize a novel semi-trapezoidal polymer, using the commercially available 9,9-dimethylxanthene and Isatin as monomers [86]. Following a quaternization process, QPDI-a AEMs were obtained. The conductivity of the QPDI-100 reached 205 mS cm^−1^ at 80 °C. The power density of H_2_−O_2_ fuel cells at 60 °C can reach 437.7 mW cm^−2^.

TB polymers can also be used as AEM membrane materials. Xu’s group first reported AEMs derived from quaternized TB-containing PIMs, including flexible DMDPM-QTB, intermediate flexible DMBP-QTB, and rigid Trip-QTB [87]. After immersion in 2 M NaOH solutions at 60 °C for 240 h, DMDPM-QTB and DMBP-QTB showed no degradation, while Trip-QTB degraded a lot due to its much greater intrinsic microporosity and thus much higher water uptake. DMBP-QTB exhibits higher temperature dependence on OH^−^ conductivity than DMDPM-QTB due to its greater rigidity, which requires a higher energy barrier for OH^−^ hopping. DMBP-QTB demonstrated a high OH^−^ conductivity of 164.4 mS cm^−1^ at 80 °C under a low ion exchange capacity of 0.82 mmol g^−1^. Nevertheless, the fragility of the TB-polymer-based membranes impeded their large-scale development. Hu et al. used a long, flexible multi-cation agent to crosslink the copolymers DPM/DMBP-TB, which were synthesized from monomers 4,4′-diaminodiphenylmethane (DPM) and DMBP [88]. The long-chain multi-cation crosslinker not only formed continuous ion-conducting channels, but also improved the membrane’s mechanical properties. The membrane with a crosslinking ratio of 1.5 exhibited a high OH^−^ conductivity of 104 mS cm^−1^ (80 °C) at a low IEC of 1.67 meq·g^−1^. Moreover, the membrane achieved a peak power density of 158 mW cm^−2^ in a single fuel cell at a current density of 330 mA cm^−2^ at 60 °C. Liu’s group introduced poly (crown ether)s into TB polymers (Tb-PCEs), attempting to improve the solubility and flexibility of TB polymers [89]. The ligands formed by the alkaline cation-binding cavities significantly enhanced the polymers’ alkaline stability and OH^−^ conductivity. The resulting AEM exhibited a high OH^−^ conductivity of 141.5 mS cm^−1^ and a peak power density of 202 mW cm^−2^ at 80 °C and a low IEC value of 1.23 mmol g^−1^. Zhang’s group also introduced the crown ether unit into a quaternized TB polymer (QTB-PCE-2.5) to improve the chemical stability of its cations, and a highest conductivity of 91.4 mS cm^−1^ at 80 °C was obtained [90]. In addition, it achieved a peak power density of 195.8 mW cm^−2^. The present results show that fabricating AEMs with a TB unit and crown ether unit is favorable for improving their performance.

Meanwhile, some new types of TB-based AEMs have also been reported. Du et al. reported a new TB-based polymer that was soluble in polar solvents like NMP and DMSO [91]. Synthesized from DMBP and 4-(2-hydroxyethoxy)-1,3-phenylenediamine dihydrochloride (HEPD), the copolymer formed a hydrogen bond network via HEPD’s terminal hydroxyl groups, which enhanced its solubility and conductivity. This copolymer can be fabricated into PEMs or grafted with cationic groups to produce AEMs. At 80 °C, the proton conductivity of HEPD/DMBP-TB and the OH^−^ conductivity of the HEPD/DMBP-QTB reached 58.3 and 105.9 mS cm^−1^, respectively. Additionally, a single cell assembled with the HEPD/DMBP-QTB AEM delivered a peak power density of 76.6 mW cm^−2^ in CO_2_-free H_2_/air at 60 °C. Liu’s group introduced a hyperbranched structure in TB AEMs, and this hyperbranched structure (QA-BTB-x%) enhanced their free volume, which improved their water uptake and thus promoted the transport of OH^−^ [92]. The membrane with a branching agent content of 5% reached a maximum OH^−^ conductivity of 95.2 mS cm^−1^ at 80 °C. The maximum power density of the assembled single cell reached 548 mW cm^−2^ in H_2_/O_2_.

PIM-based membranes can offer high ionic conductivity and selectivity in RFBs, PEMFCs and AEMFCs. The key performance parameters of PIM-based membranes in electrochemical energy storage devices are summarized in Table 3.

## 4. Current Challenges

Although the above studies demonstrate the significant potential of PIM-based membranes for ion separation, their practical implementation in ion separation still faces some challenges, as discussed below.

### 4.1. Aging of PIMs

The physical aging of PIMs remains a core obstacle. Aging has been reported to cause a gradual decline in gas separation efficiency over time for PIM membranes. However, aging behavior has not been reported in ion separation, and thus, this issue should also be considered in ion separation. Insights from gas separation studies can offer important guidance for ion separation research. Therefore, some studies about physical aging in gas permeability are displayed in this part.

The rate and extent of the aging process are influenced by complex factors, which make accurate evaluation of PIM films’ long-term stability particularly challenging. For example, testing history can greatly affect aging. Samples tested immediately after methanol treatment exhibited significantly faster performance degradation compared with those stored prior to testing [93]. Film thickness also impacts the aging rate. Harms et al. demonstrated that for films thinner than 1 µm, the aging process is nearly complete within approximately 3 months, with free volume void sizes across the entire film depth reduced to low levels. In contrast, for thicker films, aging persists for several months, exhibiting a pronounced free volume gradient from the surface to the interior [94]. In addition, the inherent structure of the PIM polymer also matters. Wang et al. suggested that while rigid molecular units enhance initial performance, they often accelerate aging. In addition, different PIM polymers may have distinct structural evolution patterns during aging [95]. Liu et al. conducted a comparative study between PIM-1 and PIM-C1, which features a spiroindene locking structure [96]. After six months of aging, both materials exhibited nearly identical total CO_2_ permeability loss (approximately 76% for PIM-1 and 77% for PIM-C1). However, the underlying mechanisms differ: PIM-1 demonstrates contraction of its micropore diameters during aging, which paradoxically enhances gas diffusion selectivity. In contrast, PIM-C1’s spiroindene units “lock” the twist angles of polymer chains, forming a more rigid framework that effectively maintains stable micropore diameters throughout the aging process.

Several strategies have been developed to delay aging, such as chemical crosslinking and filler incorporation. For instance, glyceride-crosslinked PIM-Trip-TB has been proven to effectively reduce physical aging rates [95]. In addition, filler incorporation emerges as a primary research strategy to address the physical aging issue. Early studies demonstrated that simple physical blending yielded limited improvements. For instance, Bushell et al. blended porous organic cage CC3 molecules with PIM-1, which significantly enhanced initial permeability. However, the permeability declined sharply by 60–68% within the first 300 days. This highlights the critical importance of strong interfacial interactions [97]. Wang et al. employed amine-functionalized tricyclic porous organic polymer (TP-FC-CH_2_NH_2_) as a filler, leveraging the chemical interactions to optimize interfacial compatibility. During a 210-day test, the optimal MMM formulation exhibited only a 22% reduction in CO_2_ permeability [98]. Lau et al. achieved a breakthrough by introducing the porous aromatic framework PAF-1, whose extensive aromatic structure deeply interpenetrates with the PIM-1 segment, effectively suppressing the aging process. In tests lasting over 240 days, the corresponding MMM demonstrated merely a 7% loss in CO_2_ permeability [99]. An alternative approach involves utilizing “homologous” fillers. Tamaddondar et al. developed a low-crosslink density network PIM-1 (LCD-network-PIM-1), which was chemically grafted onto a linear PIM-1 matrix. After 160 days of aging, the permeability loss of the MMMs was reduced to 29%. However, when highly crosslinked dense PIM-1 networks were used as fillers, they failed to effectively suppress aging. These studies demonstrate that the long-term stability of MMMs is collectively determined by the chemical properties of the fillers, their pore structure, surface functional groups, and the strength of their interfacial interactions with the polymer matrix [100].

Table 4 summarizes the permeance reduction in PIM-1 and some modified PIM-1 membranes in gas separation, which may provide some guidance for aging behavior investigation and aging inhibition strategy development for ion separation.

### 4.2. Trade-Off Between Mechanical Property and Ion Conductivity

The intrinsic microporosity of PIMs derives from the rigid, twisted architecture of their molecular chains. While this structural feature inhibits the dense packing of molecular chains, it also results in weak intermolecular interactions due to the absence of entanglement between flexible chain segments. Consequently, PIM-based membranes are brittle [101], generally exhibiting low mechanical strength (e.g., tensile strength and elongation at break). In practical applications, mechanical stress during membrane module assembly and pressure fluctuations during separation processes are prone to causing membrane rupture. This issue is particularly pronounced for ultra-thin PIM membranes, where mechanical stability is even harder to guarantee, ultimately limiting their application scope.

Incorporation of another polymer into PIM backbones may enhance the membrane’s mechanical properties. In Hossain et al.’s work, a PIM-PI-1 homopolymer membrane had a tensile strength of only 68.8 MPa. They performed a one-step preparation of random-type copolymers between PIM-polyimide (PIM-PI) and 4,4′-hexafluoro-isopropylidene di-phthalic anhydride (6FDA)-durene-based PI [102]. The resulting membrane had an enhanced tensile strength of 71.1–82.7 MPa.

**Figure 12:**
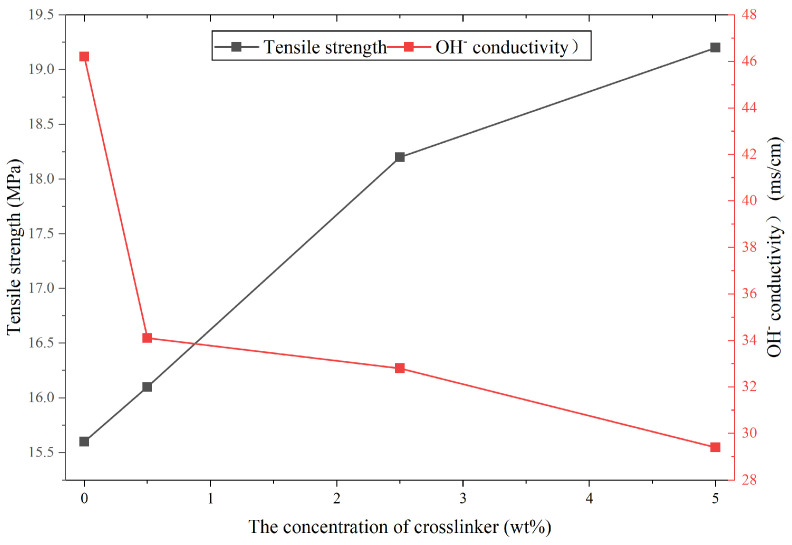
The effect of crosslinking degree on the tensile strength and ion conductivity of the crosslinked QPIM-1 membranes; the data were obtained from reference [103].

Non-crosslinked PIM membranes are confronted with a swelling issue. Crosslinking is a mainstream method to effectively enhance mechanical stability and suppress swelling by restricting polymer chain conformation [83]. Qiu et al. introduced a rigid diazide crosslinker into a quaternary ammonium-modified PIM membrane (QPIM-1), successfully preparing a rigid crosslinked PIM membrane (cQPIM-1) [103]. The results showed that crosslinking significantly improved mechanical properties. Compared to the uncrosslinked membrane, cQPIM-1’s tensile strength was increased. This is because the crosslinking reaction of the azide groups formed a covalent-bonded crosslinked network among the polymer chains, enhancing inter-chain interactions. However, crosslinking may make more distorted ion channels, decrease the connectivity of ion channels, or even block the channels, which causes a significantly weakened ion migration rate. Therefore, crosslinking hinders ion conduction to some extent, and there exists a trade-off between mechanical properties and ion conductivity. The effect of the crosslinking degree on these two parameters is shown in Figure 12, and the data were obtained from the reference.

Future efforts to improve the mechanical properties of PIM-based membranes may focus on copolymerization with flexible chain segments or crosslinking reactions to strengthen intermolecular interactions. However, a well-known trade-off between mechanical robustness and ion conductivity often exists in such materials. Therefore, the effect of crosslinking on membrane performance requires further systematic investigation, and an optimal crosslinking degree must be carefully determined to balance these key properties.

### 4.3. Other Challenges Need to Be Considered

#### 4.3.1. Complex Large-Scale Production Process

PIM-based polymers involve multi-step synthesis and purification procedures. Moreover, membrane preparation predominantly relies on solution casting, which entails dissolving polymers in suitable solvents followed by casting to form membranes. However, this casting method is afflicted by several bottlenecks. First, solvent selection is restricted to a few high-boiling polar solvents (e.g., chloroform). Such solvents exhibit high toxicity and pose significant challenges in solvent recovery, leading to high environmental costs and safety hazards during large-scale production. Second, solution casting requires slow solvent evaporation to yield a uniform membrane structure, leading to a low film-forming efficiency. Additionally, it is challenging to prepare large-area, ultra-thin continuous membranes via this approach.

#### 4.3.2. Difficulty in Regulating Surface Hydrophilicity

The molecular structure of PIMs is predominantly composed of rigid units containing hydrophobic groups, thus endowing pure PIM-based membranes with an inherently hydrophobic surface. This hydrophobicity gives rise to two key issues in aqueous separation scenarios (e.g., seawater desalination, aqueous-phase ion separation): compromised water flux and high susceptibility to membrane fouling. Currently, there are limited approaches for hydrophilic modification, and the modification process may disrupt the intrinsic microporous structure of the membranes. Therefore, the exploration of more efficient hydrophilic modification approaches is imperative for future research.

#### 4.3.3. Alkaline Stability Limitations

Many of the explored PIMs still contain vulnerable sites (e.g., benzyl quaternary ammonium groups, nitrile groups), which exhibit poor alkaline stability under high-pH conditions [84]. Therefore, more sophisticated molecular design strategies tailored to enhance alkaline stability are highly desirable for extending PIM applications in alkaline environments (e.g., fuel cells).

## 5. Conclusions

PIM-based membranes featuring continuous interconnected pore channels that fall within the microporosity range have emerged as a promising category of ion separation material. This review systematically summarizes the structural characteristics, modification strategies, and recent advances of PIM-based membranes for ion separation. These membranes demonstrate extensive application prospects across multiple critical fields, including ion resource recovery, water treatment, and electrochemical energy storage systems. By comprehensively outlining the current research progress and highlighting the existing challenges, this work aims to offer meaningful guidance for researchers in the related research fields, thus promoting the rational design, performance optimization and practical implementation of PIM-based membranes for advanced ion separation in the future.

## Figures and Tables

**Table 1:** Performance summary of PIM membranes used in ion resource recovery.

Applications	Membranes	Permeability *	Selectivity	Ref
Acid recovery	PIM-Br/1,4-Diazabicyclo [2.2.2] octane	37.00	54.3	[21]
	PIM-Br/4,4′-bipyridine	51.07	52.16	[32]
	QTB-TAPT	22.80	723.8	[35]
Alkali recovery	QA-TBF_70	300.00	181.0	[34]
	TPA100	23.00	260	[36]
Li^+^/Mg^2+^ separation	TPA100	46.20	157	[36]
	Crown-ether modified TB	13.00	35.8	[38]
	DMBP-QTB (quaternized)	2.00	152.2	[40]

* For acid recovery experiments, permeability refers to the proton transport rate, quantified as U_H_^+^ with unit of ×10^−3^ m h^−1^; for alkali recovery experiments, permeability refers to the hydroxide ion transport rate, with unit of mmol m^−2^ h^−1^; for Li^+^/Mg^2+^ separation experiments, permeability is reported as the molar flux of Li^+^, with unit of mmol m^−2^ h^−1^.

**Table 2:** Performance summary of PIM membranes used in water treatment.

Process	Membrane	Water Permeability (LMH bar^−1^)	Salt or Dye Rejection	Ref.
RO	MPDTrip	9.2	96% NaCl	[47]
	TP-DATB	2.3	99.58% NaCl	[50]
	AO-PIM-1	1.92 × 10^−4^	98% NaCl	[47]
	a-LPIM-1/MPD	62.8	97.6% NaCl	[48]
NF	PC-PIM-1	13.3	77.38% MgSO_4_	[51]
	PIM-COOH	0.86	~91% MgSO_4_	[52]
	cPIM-COOH-350	~2	~90% MgSO_4_	
	cPIM-COOH-600	~3.3	~88% MgSO_4_	
	cPIM-COOH-1100	3.83	~82% MgSO_4_	
	TTSBI/PIP-TMC	9.9	98% Na_2_SO_4_	[53]
	β-CD-PIM	15.3	95.1% Na_2_SO_4_	[54]
	PIM-1-coated UiO-66 TTSBI-TMC TTSBI-GC	~45	>95% Congo red	[55]
		480.5	89.4% Congo red	[56]
		402.4	98.8% Congo red	
	TTSBI-SDC	~100	99.0% Congo red	
	PA	13.8	99.3% Na_2_SO_4_	[57]
	PIM-1(M-3)	24.2	98.5% Na_2_SO_4_	
	PIM-CONH_2_(M-N)	28.2	94.5% Na_2_SO_4_	
	PIM-COOH(M-C)	21.0	95.0% Na_2_SO_4_	
	TBDA-DMSO	5.4	93.8% Na_2_SO_4_	[59]
	TBDA/PIP-DMSO	14.4	99.3% Na_2_SO_4_	[60]
	DATB-based NF	6.3	98.1% Na_2_SO_4_	[61]
	TBD-TMC	17.0	91% Na_2_SO_4_	[62]
	TB-PA	18.5	98.3% Na_2_SO_4_	[63]
	TTSBI/IPC	250.0	10% NaCl	[64]

**Table 3:** Summary of PIMs in electrochemical energy storage.

Application	Membrane	Conductivity (mS cm^−2^)	Performance *	Ref.
RFB	AO-PIM	-	99.00	[70]
	AO-PIM-1		99.80	[67]
	cPIM-Ph		99.90	[18]
	P/cPIM		98.00	[71]
	DMDPM-TB^+^		99.99	[39]
	DMBP-TB^+^ Q-TBF-TPB PIM-EA-TB		99.99	
			99.00	[33]
			99.80	[67]
PEMFC	OPBI/L-PIM-1	313.00	65.40	[74]
	PIM-PBIs	90.11	90.00	[75]
	NPBI/PIM-BM-15/PA	94.82	92.00	[76]
	PIM-1/PIM-Py-50	42.02	74.13	[77]
	PIM-1/PIM-MePi-50	37.11	68.52	
	CIMPIM 4-CIMPIM	330.30	86.00	[78]
		157.40	83.00	
AEMFC	DMBP-TB/50%PEKC /149%PA	61.00	-	[80]
	PIM/PSF blocks	52.60	270.00	[82]
	QPIM-1	57.00	-	[85]
	QP(SBI/AES)-x	110.00	437.00	[67]
	QPDI-a	205.00	437.70	[86]
	DMBP-QTB	164.40	-	[87]
AEMFC	DPM/DMBP-TB	104.00	158.00	[88]
	Tb-PCE-1	141.50	202.00	[89]
	QTB-PCE-2.5	91.40	195.80	[90]
	HEPD/DMBP-TB HEPD/DMBP-QTB QA-BTB-x%	58.30	-	[91]
		105.90	76.60	
		95.20	548.00	[92]

* For RFB experiments, performance refers to the Coulombic efficiency, with the unit of %; for PEMFC experiments, performance refers to the PA retention rate, with the unit of %; for AEMFC experiments, performance is reported as the Peak power density, with the unit of mW cm^−2^.

**Table 4:** Permeance reduction in PIM-1 membranes and some modified PIM-1 membranes used in gas separation.

	Membrane	Gas Permeance Loss (%)	Test Duration (Days)	Ref.
Original PIM-1	PIM-1	76.0	180	[96]
Modified PIM-1	PIM-C1	77.0	180	[96]
	CC3/PIM-1	60.0–68.0	300	[97]
	TP-FC-CH_2_NH_2_/PIM-1	22.0	210	[98]
	PAF-1/PIM-1	7.0	240	[99]
	LCD-network-PIM-1	29.0	160	[100]

## Data Availability

No new data were created or analyzed in this study.
